# Variation in hepatitis B immunization coverage rates associated with provider practices after the temporary suspension of the birth dose

**DOI:** 10.1186/1471-2431-6-31

**Published:** 2006-11-13

**Authors:** Nancy D Lin, Ken Kleinman, K Arnold Chan, Xian-Jie Yu, Eric K France, Feifei Wei, John P Mullooly, Steven Black, David K Shay, Margarette Kolczak, Tracy A Lieu, the Vaccine Safety Datalink Team

**Affiliations:** 1 Department of Ambulatory Care and Prevention, Harvard Pilgrim Health Care and Harvard Medical School, Boston, MA, USA; 2 Center for Health Policy and Center for Primary Care and Outcomes Research, Stanford University, Stanford, CA, USA; 3 Department of Epidemiology, Harvard School of Public Health, Boston, MA, USA; 4 Kaiser Permanente Colorado, Denver, CO, USA; 5 HealthPartners Research Foundation, Minneapolis, MN, USA; 6 Center for Health Research, Kaiser Permanente, Portland, OR, USA; 7 Vaccine Study Center, Kaiser Permanente Northern California, Oakland, CA, USA; 8 Centers for Disease Control and Prevention, Atlanta, GA, USA

## Abstract

**Background:**

In 1999, the American Academy of Pediatrics and U.S. Public Health Service recommended suspending the birth dose of hepatitis B vaccine due to concerns about potential mercury exposure. A previous report found that overall national hepatitis B vaccination coverage rates decreased in association with the suspension. It is unknown whether this underimmunization occurred uniformly or was associated with how providers changed their practices for the timing of hepatitis B vaccine doses. We evaluate the impact of the birth dose suspension on underimmunization for the hepatitis B vaccine series among 24-month-olds in five large provider groups and describe provider practices potentially associated with underimmunization following the suspension.

**Methods:**

Retrospective cohort study of children enrolled in five large provider groups in the United States (A-E). Logistic regression was used to evaluate the association between the birth dose suspension and a child's probability of being underimmunized at 24 months for the hepatitis B vaccine series.

**Results:**

Prior to July 1999, the percent of children who received a hepatitis B vaccination at birth varied widely (3% to 90%) across the five provider groups. After the national recommendation to suspend the hepatitis B birth dose, the percent of children who received a hepatitis B vaccination at birth decreased in all provider groups, and this trend persisted after the policy was reversed. The most substantial decreases were observed in the two provider groups that shifted the first hepatitis B dose from birth to 5–6 months of age. Accounting for temporal trend, children in these two provider groups were significantly more likely to be underimmunized for the hepatitis B series at 24 months of age if they were in the birth dose suspension cohort compared with baseline (Group D OR 2.7, 95% CI 1.7 – 4.4; Group E OR 3.1, 95% CI 2.3 – 4.2). This represented 6% more children in Group D and 9% more children in Group E who were underimmunized in the suspension cohort compared with baseline. Children in the reversal cohort in these groups remained significantly more likely to be underimmunized compared with baseline. In contrast, in a third provider group where the typical timing of the third dose was unchanged and in two other provider groups whose hepatitis B vaccination schedules were unaffected by the birth dose suspension, hepatitis B vaccination coverage either was maintained or improved.

**Conclusion:**

When the hepatitis B birth dose was suspended, provider groups that moved the first dose of vaccination to 5–6 months of age or later had decreases in hepatitis B vaccine coverage at 24 months. These findings suggest that as vaccine policy changes occur, providers could attempt to minimize underimmunization by adopting vaccination schedules that minimize delays in the recommended timing of vaccine doses.

## Background

In 1999, the Food and Drug Administration determined that cumulative exposure to mercury through vaccination exceeded one of three existing federal safety guidelines for methylmercury exposure for some children[1,2]. While information was limited regarding the potential adverse effects of exposure through childhood vaccination to thimerosal, a preservative in vaccines that is metabolized into ethylmercury, [1,2] the American Academy of Pediatrics (AAP) and the United States Public Health Service (US PHS) issued a Joint Statement in July 1999 to describe several key actions intended to assure the replacement of thimerosal-containing vaccines with thimerosal-free formulations while encouraging the maintenance of childhood vaccination coverage levels[3]. In this Statement, they recommended as an additional precaution the temporary deferment of the first dose of hepatitis B vaccine until 2–6 months of age for infants born to hepatitis B surface antigen-negative women. Specific recommendations for timing of the second and third doses in the hepatitis B vaccine series were not provided. By mid-September 1999, the first thimerosal-free hepatitis B vaccine was licensed in the United States, and a second statement was issued recommending the reinstatement of hepatitis B birth vaccination practices in hospital nurseries[4].

The rapid reversal in the hepatitis B birth dose recommendations may have led to unanticipated disruptions in immunization delivery. Timing of the first dose of hepatitis B vaccine had previously been associated with a child's up-to-date status at later ages[5]. While hospital nurseries quickly discontinued routine administration of a hepatitis B vaccine birth dose following the July 1999 Joint Statement, reinstatement of birth vaccination policies had failed to reach pre-July 1999 levels one year after the new thimerosal-free hepatitis B vaccine first became available [6-8]. In addition, a disruption in hepatitis B vaccination coverage following the birth dose suspension was observed in a nationally representative sample of 19-month olds[9].

It is unknown whether pediatricians' decisions about how to shift the recommended ages of the hepatitis B doses affected the likelihood that their patients would fail to complete this vaccine series. Understanding how different adjustments to childhood immunization recommendations may affect vaccine series completion, when changes are needed, is important because such changes have become more frequent in recent years due to vaccine shortages and new vaccines. Our study was designed to address this key issue by evaluating how the hepatitis B vaccine birth dose suspension affected immunization coverage rates among the patients of five large provider groups with varying vaccination policies both before and after the suspension.

## Methods

### Study population

This study included the membership of five large provider groups: Harvard Vanguard Medical Associates (Boston, MA), HealthPartners (Minneapolis, MN), Kaiser Permanente of Colorado (Denver, CO), Kaiser Permanente of Northern California (Oakland, CA), and Kaiser Permanente Northwest (Portland, OR). These sites participate in the Centers for Disease Control and Prevention Vaccine Safety Datalink Project, in which individual-level vaccination, demographic, and medical data are shared to facilitate vaccine safety and other vaccine-related epidemiologic research [10-12].

We studied infants who were born between October 1, 1996 and December 31, 1999, who were continuously enrolled in one of the five provider groups from birth through their second year of life, and who received at least one polio vaccination. The continuous enrollment criteria was applied to ensure that the most complete immunization history was available, while receipt of polio vaccination was used as an indicator that a child received immunizations that were recorded in the provider group information systems. The study protocol was approved by the institutional review boards at the participating provider groups and the Centers for Disease Control and Prevention.

### Definition of exposure and outcome

Each child was assigned to one of three exposure birth cohorts, based on the timing of their birth relative to the issuance and subsequent reversal of the thimerosal-related birth dose suspension recommendations. Children born between October 1, 1996 and June 30, 1999 were grouped in the "baseline" cohort. Children born between July 1, 1999 and September 30, 1999 were assigned to the birth dose "suspension" cohort, the period when the thimerosal-related recommendations were in effect[4]. Children born between October 1, 1999 and December 31, 1999 were grouped in the hepatitis B policy "reversal" cohort, denoting a period following withdrawal of the birth dose suspension recommendations.

The outcome, underimmunized for the hepatitis B vaccine series, was defined as the receipt of fewer than the three recommended doses of hepatitis B vaccine at 24 months of age (yes/no). Vaccinations that were administered before the minimum recommended age or earlier than the minimum recommended between-vaccination interval, allowing for a four-day grace period[13,14], were considered to be invalid. Only the remaining vaccinations for eligible individuals were included in our analysis.

### Statistical analysis

**Figure 1:**
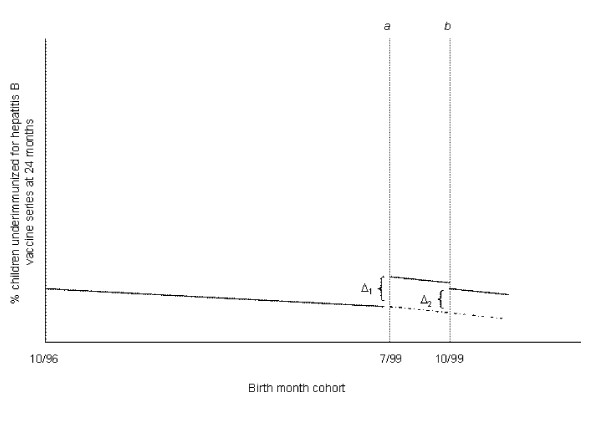
**Calculation of predicted absolute differences in percent of 24-month-olds underimmunized for the hepatitis B vaccine series, birth dose suspension and reversal periods versus baseline**. --- : % children underimmunized based on logistic regression model and the observed data. - - - - : % children underimmunized predicted from the baseline. ***Time point a***: July 1999 birth cohort, start of the birth dose suspension period. **Δ_1 _**compares % children underimmunized for the July 1999 birth cohort based on the fitted data to that predicted from the baseline. ***Time point b***: October 1999 birth cohort, following reversal of the birth dose suspension. **Δ_2 _**compares % children underimmunized for the hepatitis B vaccine series for the October 1999 birth cohort based on the fitted data to that predicted from baseline.

To evaluate the association between the hepatitis B policy reversals and hepatitis B underimmunization status at age 24 months, odds ratios and 95% confidence intervals (CI) were calculated from logistic regression models. A linear trend based on birth month cohort was included to adjust for temporal trend. We also tested whether temporal trend varied by policy period; interaction by policy period was not statistically significant, and these additional terms were not included in the final model. To aid interpretation of the logistic regression results, predicted probabilities were output from the logistic regression model for two time points (July 1999 and October 1999 birth cohorts). For each time point, the absolute difference in hepatitis B underimmunization at age 24 months was calculated by comparing the percent of children underimmunized as fitted from the regression model and observed data, to the predicted percent underimmunized based on extrapolation from the pre-suspension baseline cohort of children born between October 1996 – June 1999 (i.e., absolute difference = [fitted % underimmunized] - [predicted % underimmunized]) (Figure 1).

All analyses were performed using SAS software, Version 8.2 of the SAS System for Windows (SAS Institute, Cary, NC).

## Results

### Study population

Of the 214,499 children born and enrolled at birth in the five provider groups during the study period, 87,447 were continuously enrolled through their 24-month birthday. Of these children, 85,064 (97.3%) received at least one polio vaccination. Preliminary data audits revealed two subgroups of children for whom immunization histories were potentially incomplete: (1) children who were born during a potential disruption in the immunization tracking system during the early baseline period in one provider group (Group B) (n = 4,112) and (2) children in a second provider group (Group C) who received vaccinations in a set of outpatient clinics for which information on health care utilization was identified as likely to be incomplete (n = 651). Exclusion of these children resulted in a final population of 80,301 children.

### Variation in hepatitis B birth vaccination practices

**Figure 2:**
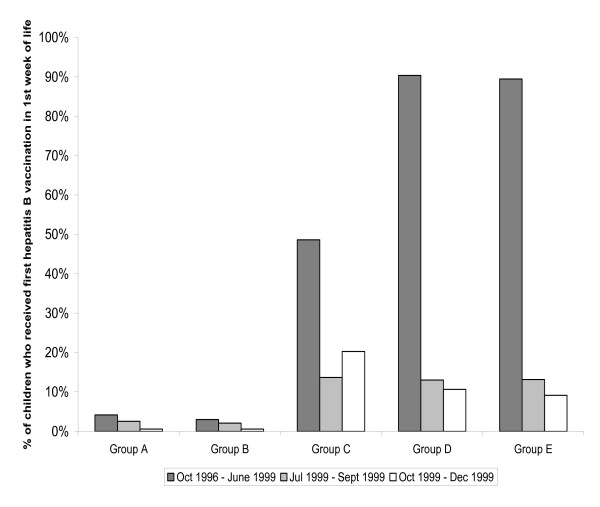
**Percent of children enrolled in five large U.S. provider groups who received a first hepatitis B vaccination within the first week of life, October 1996 – December 1999**. Baseline cohort: born between October 1996 – June 1999 (n = 67,835). Birth dose suspension cohort: born between July 1999 – September 1999 (n = 6,401). Reversal cohort: born between October 1999 – December 1999 (n = 6,065).

Prior to the birth dose suspension, administration of hepatitis B vaccination within the first week of life ("birth" vaccination) varied widely across the provider groups (Figure 2). While 4% of children in Group A and 3% in Group B received a hepatitis B birth dose, 49% of children in Group C, 90% in Group D, and 89% in Group E did so. After the birth dose suspension, children in all five provider groups were less likely to receive a hepatitis B birth dose, with children in Group D and Group E experiencing substantial declines that persisted even after reversal of the suspension recommendations (baseline vs. suspension vs. reversal Group D: 90% vs. 13% vs. 11%; Group E: 89% vs. 13% vs. 9%).

### Impact of the birth dose suspension on failure to complete the hepatitis B vaccine series by 24 months

In Groups A, B, and C, the percent of children who were underimmunized for the hepatitis B vaccine series at 24 months remained similar or decreased following the birth dose suspension (Group A: 6% baseline vs. 5% suspension vs. 7% reversal, χ^2 ^p = 0.42; Group B: 11% vs. 5% vs. 4%, χ^2 ^p < 0.0001; Group C: 12% vs. 10% vs. 9%, χ^2 ^p < 0.0001)(Table 1). In contrast, children were generally more likely to be underimmunized for the hepatitis B vaccine series at 24 months following the birth dose suspension in Group D (7% baseline vs. 9% suspension vs. 8% reversal, χ^2 ^p = 0.09) and in Group E (9% baseline vs. 14% suspension vs. 11% reversal, χ^2 ^p = 0.0002).

**Table 1:** Change in practices for the hepatitis B birth dose and underimmunization at age 24 months

	Absolute difference in % children who received 1^st ^hepatitis B vaccination during first week of life		% underimmunized for hepatitis B vaccine series at age 24 months, unadjusted results		
	
	Suspension vs. Baseline	Reversal vs. Baseline	Baseline	Suspension	Reversal
Group A	-2%	-4%	6%	5%	7%
Group B	-1%	-2%	11%	5%	4%
Group C	-35%	-28%	12%	10%	9%
Group D	-77%	-79%	7%	9%	8%
Group E	-76%	-80%	9%	14%	11%

"Birth vaccination" is defined as receipt of a hepatitis B vaccination between 0 and 7 days of age. Baseline cohort: Born between October 1996 – June 1999 (n = 67,835). Birth dose suspension cohort: Born between July – September 1999 (n = 6,401). Reversal cohort: Born between October – December 1999 (n = 6,065).

Adjusting for temporal trend in the logistic regression analysis yielded similar findings to the unadjusted results (Table 2). In Provider Group A, immunization coverage among 24 month olds remained similar before and following the hepatitis B birth dose suspension recommendations. In Provider Groups B and C, children were less likely to be underimmunized in both the suspension period (Table 2) and the reversal period (data not shown) compared to the pre-suspension baseline. In contrast, children in Group D and Group E were significantly more likely to be underimmunized for the hepatitis B series at 24 months during the suspension period (Table 2), representing 6% more children in Group D and 9% more children in Group E who were underimmunized for hepatitis B vaccine in the July 1999 birth dose suspension cohort compared to that predicted from baseline. Children in these two provider groups remained significantly more likely to be underimmunized in the reversal period (Group D OR = 2.5, 95% CI: 1.4 – 4.5; Group E OR = 2.7, 95% CI: 1.9 – 3.9), representing 5% more children in Group D and 7% more children in Group E who were underimmunized in the October 1999 birth cohort compared to that predicted from baseline.

**Table 2:** Typical age(s) at receipt of hepatitis B vaccine doses and change in % underimmunized at 24 months for the hepatitis B vaccine series, baseline vs. birth dose suspension cohorts, children enrolled in five large U.S. provider groups, October 1996 – December 1999

	Modal Age in Months at Hepatitis B Vaccination*					
			
Group	Exposure cohort	Dose 1	Dose 2	Dose 3	Predicted Absolute Change in % Underimmunized at 24 Months in the Suspension vs. Baseline Period^†^	OR for Being Underimmunized at 24 Months in the Suspension vs. Baseline Period (95% CI)^§^
A	Baseline:	≤2	3–4	11–12	-2%	0.7 (0.4 – 1.3)
	Suspension:	≤2	3–4	11–12		
B	Baseline:	≤2	≤2	17–18	-5%^‡^	0.5 (0.2 – 0.9)
	Suspension:	≤2	3–4	11–12		
C	Baseline:	Birth	≤2	5–6	-3%^‡^	0.8 (0.7 – 0.9)
	Suspension:	≤2	3–4	5–6		
D	Baseline:	Birth	≤2	9–10	+6%^‡^	2.7 (1.7 – 4.4)
	Suspension:	5–6	9–10	15–16		
E	Baseline:	Birth	≤2	5–6	+9%^‡^	3.1 (2.3 – 4.2)
	Suspension:	5–6	9–10	11–12		

*Baseline cohort: Born between October 1996 – June 1999 (n = 67,835). Birth dose suspension cohort: Born between July – September 1999 (n = 6,401). Age at vaccination was grouped into the following categories: birth (0–7 days), ≤ 2 months (8 days – 2 months), 3–4 months, 5–6 months, 7–8 months, 9–10 months, 11–12 months, 13–14 months, 15–16 months, 17–18 months, 19–20 months, 21–24 months, and did not receive dose by 24 months. For each dose, the most common ages at vaccination were listed.

^† ^For each provider group, the predicted absolute difference was calculated comparing the % hepatitis B underimmunized at age 24 months among the July 1999 birth cohort as fitted from multivariate regression models adjusting for temporal trend versus the % underimmunized as extrapolated from baseline (i.e., absolute difference = [fitted % underimmunized]_July1999 _- [predicted % underimmunized]_July1999_).

^‡ ^p < 0.05.

^§ ^ORs are from logistic regression models adjusted for temporal trend.

### Variation in the timing of hepatitis B vaccinations

**Figure 3:**
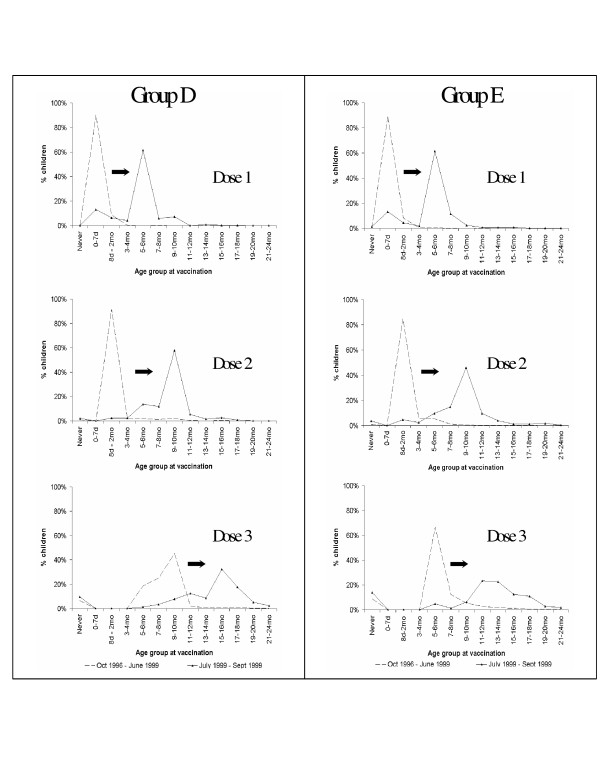
**Delays in age at receipt of hepatitis B vaccine doses among children enrolled in provider groups D and E following the hepatitis B birth dose suspension recommendations, baseline cohort vs. birth dose suspension cohort**. - - - : Baseline cohort of children born between October 1996 – June 1999 (n = 67,835). : Birth dose suspension cohort of children born between July – September 1999 (n = 6,401). Age at vaccination was grouped into the following categories: birth (0–7 days), < 2 months (8 days – 2 months), 3–4 months, 5–6 months, 7–8 months, 9–10 months, 11–12 months, 13–14 months, 15–16 months, 17–18 months, 19–20 months, 21–24 months, and did not receive dose by 24 months ("never").

Further assessment of the scheduling of hepatitis B vaccination indicated that the typical timing of all three doses in the hepatitis B vaccine series varied across the five provider groups, both during the baseline period and following the hepatitis B birth dose suspension (Table 2). In Groups A and B, where hepatitis B vaccination at birth was infrequent, children born following the birth dose suspension typically received their hepatitis B vaccine doses at similar or earlier ages compared to the baseline cohort. In Group C, where 49% of children received a hepatitis B birth vaccination but where an improvement in hepatitis B immunization coverage among 24-month-olds was observed, slight shifts toward delayed receipt of the first and second doses occurred during the suspension period; at the same time, the most common timing of the third dose remained at age 5–6 months (38% baseline vs. 31% suspension) or age 9–10 months (24% baseline vs. 28% suspension). In contrast, while 89–90% of children in Group D and Group E had received a hepatitis B vaccination at birth during the baseline period, the most common age at receipt of the first hepatitis B vaccination was shifted to 5–6 months of age during the birth dose suspension (62% in each Group). In these two provider groups, delays and less uniformity in the timing of the second and third dose of hepatitis B vaccine were also observed during the birth dose suspension (Table 2; Figure 3). Children born in these provider groups during the baseline period typically received the second dose of hepatitis B vaccine by 2 months of age (92% in Group D; 85% in Group E) and the third dose, between 7–10 months of age in Group D (70%) and 5–6 months of age in Group E (67%). Among the birth dose suspension cohort, the age at receipt of the second dose was most commonly shifted to 9–10 months and the third dose, to 11–12 months of age or later. Delays in the timing for the three hepatitis B doses in these provider groups were similar in the reversal period to that observed during the suspension period (data not shown).

## Discussion

This study found that in the populations of five large provider groups, hepatitis B vaccine coverage decreased after national recommendations to suspend the birth dose, but only in those provider groups that shifted the first dose of hepatitis B from birth to 5–6 months of age or later. These disruptions in immunization scheduling and coverage persisted after approval of the first thimerosal-free hepatitis B vaccine. In contrast, immunization coverage remained the same or improved over the same time period in settings that experienced either no change or minor shifts in the typical timing of hepatitis B vaccinations following the birth dose suspension recommendations.

Our findings may shed light on how providers can choose the optimal ways to shift the timing of vaccines when national committees make changes in the routine immunization schedule. Many vaccine schedule changes have been issued in recent years due to concerns about thimerosal, vaccine shortages, and the introduction of new vaccines [15-17]. Our findings confirm those of Luman et al.,[9] who found declines in birth vaccination as well as in national hepatitis B coverage rates among 19-month-olds associated with the birth dose suspension. Our results go beyond the previous study to suggest that disruptions in coverage were associated with local baseline policies that determined whether provider practices needed to be modified in response to the birth dose suspension, and with how different provider groups changed the timing of hepatitis B vaccine doses in response to the suspension. Major disruptions following the birth dose suspension were restricted to those sites where universal hepatitis B birth vaccination had been the dominant pre-existing practice. Furthermore, increases in hepatitis B underimmunization among 24 month olds were seen only in sites where the first dose was commonly delayed by at least 5 months. With this delay in administration of the first dose, scheduling typically observed during the baseline period for the second (≤2 months) and third (5–6 months or 9–10 months) doses could not be followed during the birth dose suspension. Provider practices at the other sites showed minimal or no downstream adjustments in immunization scheduling. Similar to Luman et al.,[9] we found no consistent patterns in immunization coverage for other childhood vaccine series in relation to the hepatitis B policy reversals (data not shown).

Some of the hepatitis B coverage changes we observed across these settings could have been due to other local setting influences. For example, two of the provider groups used a combination hepatitis B-Hib vaccine which is recommended at 2, 4, and 12–15 months of age[13]. The combination hepatitis B-Hib vaccine was available in Group A throughout the entire study period, and this provider group neither changed the timing of its doses nor experienced a significant change in hepatitis B underimmunization in response to the suspension. Group B began use of the combination hepatitis B-Hib vaccine around the time of the hepatitis B birth dose suspension. This may have contributed to the shift toward earlier ages for receipt of the third hepatitis B vaccine dose and the improved coverage rates in this group.

This study was conducted among a small sample of large provider groups, with populations that had insurance and good access to primary care. These results may not generalize to other settings, uninsured populations or groups with worse access to care. We also restricted the study population to children who were continuously enrolled during the first two years of life and had at least one polio vaccination recorded in the provider group information systems; these children are likely to have experienced less scattering of their immunization records and to have had more opportunities to catch up on immunizations. Thus, the decreases in hepatitis B coverage we observed in two of our study's provider groups might be larger in more vulnerable populations.

## Conclusion

We conclude that substantially delaying the timing of the first hepatitis B vaccination dose in response to the nationally recommended birth dose suspension may be associated with downstream disruptions in immunization coverage. This suggests that as vaccine policy changes occur, providers may be able to minimize underimmunization by adopting vaccination schedules that minimize delays in the recommended timing of vaccine doses.

## Competing interests

The author(s) declare that they have no competing interests.

## Authors' contributions

NL generated the study idea, wrote the protocol, led the team, carried out the analysis, and drafted the paper. KK participated in study design and provided biostatistical guidance. KAC and TAL obtained funding, provided supervision, participated in study design and interpretation of analyses, and revised the paper. XY participated in study design and conducted analyses. EKF, FW, JM, SB, DS, and MK participated in study design, contributed data, participated in interpretation of analyses, and helped revise the paper. All authors read and approved the final manuscript.

## Pre-publication history

The pre-publication history for this paper can be accessed here:


